# Real-world use of eravacycline for the treatment of mono- and polymicrobial infections involving Enterobacterales

**DOI:** 10.1128/spectrum.02262-25

**Published:** 2026-03-23

**Authors:** Sean R. Van Helden, Ashlan J. Kunz Coyne, Sara Alosaimy, Abdalhamid M. Lagnf, Kyle C. Molina, S. Lena Kang-Birken, Kimberly C. Claeys, Mark Biagi, Justin Andrade, Alaina DeKerlegand, Melanie Rae Schrack, Tristan Gore, Madeline A. King, Benjamin M. Pullinger, Serina Tart, Athena L. V. Hobbs, Jazmin Agee, Nicholson B. Perkins, Michael P. Veve, Reese Cosimi, Shelbye R. Herbin, Bruce M. Jones, Amy K. Feehan Curley, Chloe Judd, Mohammed Al Musawa, Michael J. Rybak

**Affiliations:** 1 Department of Pharmacy Practice, Eugene Applebaum College of Pharmacy and Health Sciences, Wayne State University 15538, Detroit, Michigan, USA; 2 University of Kentucky College of Pharmacy 15511, Lexington, Kentucky, USA; 3 University of Colorado Anschutz School of Medicine 12225 , Aurora, Colorado, USA; 4 Santa Barbara Cottage Hospital 22854, Santa Barbara, California, USA; 5 University of Maryland School of Pharmacy 15513, Baltimore, Maryland, USA; 6 UW Health SwedishAmerican Hospital 6938 , Rockford, Illinois, USA; 7 University of Illinois at Chicago 14681, Chicago, Illinois, USA; 8 Touro College of Pharmacy, The Brooklyn Hospital Center 471068 , New York, New York, USA; 9 Our Lady of the Lake Regional Medical Center 23087, Baton Rouge, Louisiana, USA; 10 College of Pharmacy, University of South Carolina 15525, Columbia, South Carolina, USA; 11 Philadelphia College of Pharmacy, Saint Joseph’s University 659977, Philadelphia, Pennsylvania, USA; 12 Cooper University Hospital 737464, Camden, New Jersey, USA; 13 Cape Fear Valley Health 3343, Fayetteville, North Carolina, USA; 14 Methodist University Hospital 23515, Memphis, Tennessee, USA; 15 University of Tennessee Health Science Center College of Pharmacy 15527, Memphis, Tennessee, USA; 16 University of Tennessee Medical Center 21823, Knoxville, Tennessee, USA; 17 Ascension St. Vincent Hospital 24104 , Indianapolis, Indiana, USA; 18 Department of Pharmacy, Henry Ford Hospital 24016, Detroit, Michigan, USA; 19 St. Joseph’s/Candler Health System 1468 , Savannah, Georgia, USA; 20 Ochsner Clinic Foundation 633467 , New Orleans, Louisiana, USA; 21 Ochsner Clinical School, The University of Queensland 589787, New Orleans, Louisiana, USA; 22 Boehringer Ingelheim 6893 , Ridgefield, Connecticut, USA; 23 Department of Pharmacy, Harper University Hospital, Detroit Medical Center 2970, Detroit, Michigan, USA; 24 Department of Medicine, Division of Infectious Diseases, School of Medicine, Wayne State University 12267, Detroit, Michigan, USA; University at Albany, Albany, New York, USA

**Keywords:** eravacycline, Enterobacterales, CRE, carbapenem-resistant Enterobacterales, antibiotic resistance, metallo-beta-lactamase, beta-lactamases, multidrug resistance, ERV, tetracycline, *Klebsiella*, *Escherichia coli*

## Abstract

Bacterial species in the Enterobacterales order are commonly encountered causative organisms in hospital-acquired infections. Furthermore, the incidence of carbapenem-resistant Enterobacterales (CRE) is a growing threat worldwide. Eravacycline (ERV) is a broad-spectrum fluorocycline antibiotic with activity against Enterobacterales, including CRE, and is approved for the treatment of complicated intra-abdominal infections (cIAI) in the United States and Europe. We conducted a subpopulation analysis of a previously published real-world study evaluating the efficacy of eravacycline for the treatment of infections involving Enterobacterales. Adult patients who received eravacycline for ≥72 h for any infection type involving an Enterobacterales organism were included. The primary outcome was clinical success, and secondary outcomes comprised 30-day all-cause and in-hospital mortality, 30-day microbiological and symptomatic recurrence, and 30- and 60-day hospital readmission. A total of 155 patients were eligible for inclusion. The primary causative organisms were *Klebsiella pneumoniae* (34.2%), *Escherichia coli* (32.9%), and *Enterobacter cloacae* (31.3%), and 23.9% were CRE. A majority of infections (77.4%) were polymicrobial. The most common infection types were intra-abdominal (34.8%), skin and soft tissue (25.2%), and pneumonia (17.4%). The predominant rationale for ERV use was consolidation of the antibiotic regimen (56.8%), and the median (interquartile range) ERV duration was 6.0 days (3.3–12.0). Clinical success was observed in 84.5% of patients. The 30-day all-cause and infection-related mortality were 13.5% and 8.4%, respectively. Microbiological recurrence was low (1.3%), and the 30-day hospital readmission rate was 16.9%. Eravacycline was well-tolerated, with 8.3% of patients experiencing treatment-emergent adverse events (TEAE) and 0.6% resulting in discontinuation of ERV.

**IMPORTANCE:**

As the incidence of infections caused by carbapenem-resistant Enterobacterales (CRE) continues to rise globally, novel therapeutic approaches are necessary to combat these difficult-to-treat infections. While β-lactams remain the mainstay of therapy for these patients, increasing rates of metallo-β-lactamase-producing organisms render nearly all β-lactam antibiotics ineffective. Furthermore, >15% of carbapenem-resistant Enterobacterales are non-carbapenemase-producing, circumventing the benefits provided by co-administration of β-lactamase inhibitors. Eravacycline (ERV) is a promising non-β-lactam antibiotic with a broad spectrum of activity, including methicillin-resistant *Staphylococcus aureus*, vancomycin-resistant Enterococci, and carbapenem-resistant Enterobacterales. Few data exist on the real-world use of eravacycline for the treatment of Enterobacterales infections specifically. In this study, we assessed the safety and effectiveness of eravacycline for the treatment of Enterobacterales infections in a real-world setting. These findings suggest that eravacycline may be a viable therapeutic agent for these infections, expanding our treatment options for multidrug-resistant (MDR) pathogens.

## INTRODUCTION

Species in the Enterobacterales order comprise two of the top five causative organisms of hospital-acquired infections in the United States, including pneumonia, surgical-site, intra-abdominal, and skin and soft-tissue infections (1). Most Enterobacterales species employ several intrinsic and acquired resistance mechanisms to evade antibiotics, including upregulation of efflux pumps, porin loss, target modification, and drug modification (such as β-lactamase production) (2). These organisms readily exchange mobile genetic elements, facilitating the horizontal transfer of antimicrobial resistance genes, leading to the proliferation of genes such as those encoding extended-spectrum β-lactamases (ESBLs), including the widely disseminated CTX-M-15 (2, 3). Many Enterobacterales species also harbor chromosomally encoded β-lactamases, including inducible AmpC β-lactamases. These enzymes may provide intrinsic, clinically relevant resistance to many β-lactam antibiotics, particularly in *Klebsiella aerogenes, Citrobacter freundii,* and *Enterobacter cloacae* (4).

Carbapenem-resistant Enterobacterales (CRE) is becoming increasingly prevalent in healthcare-associated infections and is considered an “urgent” antimicrobial resistance threat by the United States Centers for Disease Control and Prevention (CDC) and a “critical”-priority pathogen by the World Health Organization (5, 6). Carbapenem resistance in Enterobacterales is primarily driven by the production of carbapenemases, including KPC, OXA-48, and metallo-β-lactamases (7), which render most β-lactam antibiotics ineffective. These enzymes are frequently circumvented by employing novel, broad-spectrum β-lactamase inhibitors in combination with β-lactam antibiotics; however, resistance to these combinations can occur when several β-lactamase enzymes or other resistance mechanisms are concomitantly expressed (8, 9). Furthermore, studies have demonstrated that 19%–70% of CRE are non-carbapenemase-producing, instead relying on non-enzymatic mechanisms of resistance (10–12).

One strategy to bypass frequently encountered resistance mechanisms in CRE is to employ non-β-lactam antibiotics. Eravacycline (ERV) is a fluorocycline antibiotic approved for use in the United States and European Union for the treatment of complicated intra-abdominal infections (cIAI) (13, 14). ERV has a broad spectrum of activity, with efficacy against many gram-positive, gram-negative, atypical, and anaerobic bacteria (including *Clostridioides difficile*) (15). It also maintains activity against many multidrug-resistant (MDR) phenotypes, including ESBL-producing and carbapenem-resistant Enterobacterales, methicillin-resistant *Staphylococcus aureus*, and vancomycin-resistant Enterococci (15). ERV improves on previous generations of tetracycline antibiotics due in part to its evasion of tetracycline-specific efflux pumps (TetA and TetB) and its ability to circumvent ribosomal protection proteins (such as TetM), which are widely expressed among many Enterobacterales species (16). Given its broad spectrum of activity, ERV is a particularly attractive therapeutic option for cIAI, a setting in which polymicrobial infection is common (17).

The efficacy of ERV for the treatment of cIAI was established in the IGNITE1 and IGNITE4 phase III clinical trials (18, 19). IGNITE1 demonstrated non-inferiority in clinical cure compared to ertapenem for the treatment of cIAI, while IGNITE4 demonstrated non-inferiority in clinical cure compared to meropenem for the treatment of cIAI. Furthermore, clinical cure rates for ESBL-producing Enterobacterales were similar between the ERV group (87.5%) and the meropenem group (84.6%) in the IGNITE4 trial. Notably, 71% of patients in the IGNITE4 trial had polymicrobial infection. Despite favorable randomized controlled trial findings, real-world data supporting the use of ERV for the treatment of infections involving Enterobacterales are limited to small case series and pooled analyses (20, 21).

To assess the real-world efficacy and safety of ERV, our group previously conducted a multicenter, retrospective cohort study of 416 patients who received ERV ≥72 h for any organism or infection type (22). A total of 315 (75.7%) patients achieved clinical success, and 94.7% were alive at 30 days. This study characterized the real-world outcomes of patients receiving ERV across multiple organisms, prioritizing overall outcomes rather than organism-specific analyses. Therefore, we sought to characterize the real-world use of ERV for the treatment of infections involving Enterobacterales specifically, including ESBL-producing isolates and CRE, an area in which few real-world data exist. Furthermore, we sought to describe granular infection- and treatment-specific characteristics (e.g., infection sources, prior antimicrobial exposure, and treatment patterns) among patients with Enterobacterales-involved infections treated with ERV. Accordingly, we conducted a descriptive subpopulation analysis of our prior study focusing on patients who received ERV for mono- and polymicrobial infections involving Enterobacterales, including ESBL-producing isolates and CRE.

## MATERIALS AND METHODS

This was a multicenter retrospective real-world cohort study conducted across 17 hospitals in the United States evaluating the efficacy and safety of ERV for the treatment of Enterobacterales infections from October 2018 to August 2022. Patients were eligible for inclusion if they received ERV for ≥72 h for the treatment of an infection caused by at least 1 organism in the Enterobacterales order. Index cultures could be isolated from any source. Subsequent ERV courses must have been separated by at least 90 days, and patients who were pregnant or incarcerated were ineligible for inclusion. The study population was a subset of 155 patients from a previously published real-world study of 416 patients evaluating the efficacy of ERV for the treatment of any pathogen (22). Among these 155 patients, a total of 176 Enterobacterales isolates were recovered in culture as some patients had more than one Enterobacterales species identified in the index culture. This subpopulation was treated across 17 of the 19 hospitals included in the parent study. The primary outcome for this study was clinical success, defined as the resolution or improvement of infectious signs and symptoms, including infection-related leukocytosis and fever, or as documented by the treating physician in clinical notes. Secondary outcomes included 30-day all-cause mortality, 30-day infection-related mortality, 30-day microbiological recurrence of primary causative organism(s), 30-day symptomatic recurrence, 30-day and 60-day hospital readmission, and incidence of treatment-emergent adverse events (TEAE). Subgroup analyses of the primary and secondary outcomes were conducted among patients with ESBL-producing isolates, CRE, and non-ESBL, non-CRE isolates. Organism-specific outcomes were reported for any Enterobacterales species with at least 20 observations.

Deidentified data were collected from the electronic health records of each participating site and were recorded using Research Electronic Data Capture (REDCap) electronic data capture tools hosted at Wayne State University (23, 24). REDCap is a secure, web-based software platform designed to support data capture for research studies. The study methodology was approved by the institutional review board at each participating site in addition to the host site (Wayne State University) and was conducted in accordance with the ethical principles outlined in the Declaration of Helsinki. Statistical methods were descriptive in nature and comprised frequencies and appropriate measures of centrality and variability and were performed using SPSS Statistics software version 30.0 (IBM Corp., Armonk, NY, USA) (25). All antibiotic susceptibilities were determined using breakpoints established by the Clinical & Laboratory Standards Institute (CLSI) M100 (ED34) (26). If breakpoints were not available from the CLSI, breakpoints established by the United States Food and Drug Administration (FDA) were utilized to determine susceptibility (27). Illness severity was assessed via the Acute Physiology and Chronic Health Evaluation II (APACHE II) score (28). Comorbidity burden was determined using the Charlson Comorbidity Index (CCI) (29).

## RESULTS

A total of 155 patients were eligible for inclusion in this analysis. Baseline characteristics are described in Table 1. Patients had a mean age of 56.7 years, and 57% were male. A majority of patients were White (52%) or Black (35%). The median body mass index (BMI) was 27.6 (interquartile range [IQR]: 23.5–34.5) kg/m^2^, with 39% of patients classified as obese (BMI ≥30 kg/m^2^). The median APACHE II score was 14.0 (IQR: 8.0–20.0), indicating a moderate risk of in-hospital mortality. Common comorbid conditions included diabetes (40%), peripheral vascular disease (21%), heart failure (17%), and malignancy (15%). Eighteen percent of patients had moderate-to-severe chronic kidney disease, 10% had moderate-to-severe liver disease, and 12% were immunosuppressed. The median hospital length of stay was 24.5 days, and 61% of patients were admitted to the ICU at least once during their hospitalization.

**TABLE 1:** Baseline characteristics (*n* = 155)^*a*^^,^^*f*^

Parameter	Value (n, %)
Age (years) (mean, SD)	56.7 (14.3)
Male	88 (56.8)
Race/ethnicity	
White^*b*^	80 (51.6)
Black	54 (34.8)
Hispanic	17 (11.0)
Asian	3 (1.9)
Others	1 (0.6)
BMI (kg/m^2^) (median, IQR)	27.6 (23.5–34.5)
Obese (BMI ≥30 kg/m^2^)	60 (38.7)
Admission source	
Home	91 (58.7)
Transfer from an outside hospital	32 (20.6)
SNF/LTC	21 (13.5)
Referral from clinic (ID clinic, dialysis, etc.)	5 (3.2)
Inpatient rehabilitation facility	3 (1.9)
Others	2 (1.3)
Illness severity scores	
APACHE II score (median, IQR)	14.0 (8.0–20.0)
Charlson Comorbidity Index (median, IQR)	4.0 (2.0–6.0)
Comorbid conditions	
Diabetes	62 (40.0)
Peripheral vascular disease	32 (20.6)
Moderate-to-severe chronic kidney disease^*c*^	28 (18.1)
Heart failure	27 (17.4)
Malignancy	23 (14.8)
Moderate-to-severe liver disease^*d*^	16 (10.3)
COPD	15 (9.7)
History of *Clostridioides difficile*	13 (8.4)
Cerebrovascular disease	12 (7.7)
Chronic dialysis	11 (7.1)
Asthma	6 (3.9)
IV drug use	4 (2.6)
Immunosuppression^*e*^	18 (11.6)
Cytotoxic chemotherapy	6 (3.9)
High-dose corticosteroids	5 (3.2)
Neutropenia	5 (3.2)
Recent solid organ transplant (90 days)	4 (2.6)
Functional or surgical splenectomy	3 (1.9)
Recent bone marrow transplant (90 days)	1 (0.6)
Patients with MDR risk factors	124 (80.0)
≥48 h hospitalization within 90 days	85 (54.8)
≥24 h antibiotics within 90 days	78 (50.3)
Surgery ≤30 days before index culture	45 (29.0)
Prior infection with resistant organism	31 (20.0)
SNF/LTC resident	21 (13.6)
Home wound care	18 (11.6)
Colonization with resistant organism(s)	14 (9.0)
Chronic dialysis within 30 days	7 (4.5)
Home infusion	6 (3.9)
Hospital length of stay, days (median, IQR)	24.5 (12.0–43.0)
ICU admissions	
At least one ICU admission during hospitalization	95 (61.3)
Patients with >1 ICU admission during hospitalization	21 (13.5)
In ICU at index culture collection	41 (26.5)
Total ICU length of stay, days (median, IQR)	11.0 (4.3–30.8)

^*a*^ Data presented as number (%), mean (SD), or median (IQR), as appropriate.

^*b*^ Includes patients of Middle Eastern descent.

^*c*^ Kidney Disease Outcomes Quality Initiative (KDOQI) chronic kidney disease stages 3–5 or GFR < 60 mL/min or on chronic dialysis.

^*d*^ Portal hypertension or cirrhosis.

^*e*^ Immunosuppression factors: neutropenia (absolute neutrophil count <500); splenectomy (functional or surgical); high-dose corticosteroids (≥ prednisone 20 mg/day or equivalent).

^*f*^ Abbreviations:* *SD, standard deviation; BMI, body mass index; IQR, interquartile range; SNF/LTC, skilled nursing facility/long-term care facility; ID, infectious diseases; APACHE II, Acute Physiology and Chronic Health Evaluation II; COPD, chronic obstructive pulmonary disease; MDR, multidrug resistance; ICU, intensive care unit.

The most common source of infection was intra-abdominal (35%), followed by skin and soft-tissue (25%), pneumonia (17%), and osteomyelitis/septic arthritis/prosthetic joint infection (10%) (Table 2). A majority of causative organisms were *Klebsiella pneumoniae* (34%), *Escherichia coli* (33%), *Enterobacter cloacae* (21%), or *Klebsiella oxytoca* (8%), and most infections were polymicrobial (77%). Twenty-four percent of Enterobacterales isolates were resistant to at least one carbapenem (CRE), with 6% of isolates exhibiting ertapenem mono-resistance. Among patients with polymicrobial infection, Enterobacterales were most frequently co-isolated with *Enterococcus* spp., *Staphylococcus* spp., anaerobic organisms, and non-fermenting gram-negative bacilli. The most frequent co-infecting organisms were *Enterococcus faecalis* (21%), *Enterococcus faecium (19%*), *Acinetobacter baumannii-calcoaceticus* complex (12%), *Staphylococcus aureus* (8%), *Streptococcus* spp. (5%), anaerobic pathogens (5%), and *Stenotrophomonas maltophilia* (4%).

**TABLE 2:** Infection characteristics (*n* = 155)^*a*^^,^^*i*^

Parameter	Value
Culture specimen^*b*^	
Wound	37 (23.9)
Blood	37 (23.9)
Tissue	35 (22.6)
Fluid	34 (21.9)
Respiratory culture specimen	22 (14.2)
Sputum	17 (11.0)
Bronchoalveolar lavage	5 (3.2)
Urine	7 (4.5)
Abscess aspirate	3 (1.9)
Other aspirate	2 (1.3)
Bone	2 (1.3)
None	2 (1.3)
Primary source of infection	
Intra-abdominal	54 (34.8)
Skin and soft tissue	39 (25.2)
Pneumonia	27 (17.4)
Ventilator-associated^*c*^	18 (11.6)
Osteomyelitis/septic arthritis/PJI	15 (9.7)
Urinary tract	7 (4.5)
Primary bacteremia	3 (1.9)
Invasive prosthetic device	2 (1.3)
Intravenous catheter	1 (0.6)
Other	4 (2.6)
Unknown	3 (1.9)
*Enterobacterales isolated*	
*Klebsiella pneumoniae*	54 (34.2)
*Escherichia coli*	51 (32.9)
*Enterobacter cloacae*	33 (21.3)
*Klebsiella oxytoca*	12 (7.7)
*Klebsiella aerogenes*	7 (4.5)
*Citrobacter freundii*	6 (3.9)
*Proteus mirabilis^d^*	5 (3.2)
*Morganella morganii*	4 (2.6)
*Providencia stuartii^d^*	3 (1.9)
*Serratia marcescens*	3 (1.9)
*Citrobacter koseri*	2 (1.3)
*Citrobacter brakkii*	1 (0.6)
*Cronobacter spp*.	1 (0.6)
*Proteus vulgaris^d^*	1 (0.6)
*Pluralibacter gergoviae*	1 (0.6)
*Serratia liquefaciens*	1 (0.6)
Resistance characteristics	
ESBL^*e*^	31 (20.0)
CRE^*f*^	37 (23.9)
Ertapenem mono-resistant	9 (5.8)
Eravacycline susceptibility^*g*^ (*n* = 11)	
Susceptible	7 (63.6)
Resistant	4 (36.4)
Tigecycline susceptibility^*e*^ (*n* = 66)	
Susceptible	59 (89.4)
Intermediate	2 (3.6)
Resistant	5 (9.1)
Additional pathogens treated with ERV	
*Enterococcus faecalis*	32 (20.6)
*Enterococcus faecium*	29 (18.7)
*Acinetobacter baumannii-calcoaceticus* complex	18 (11.6)
*Staphylococcus aureus*	13 (8.4)
Methicillin-resistant *Staphylococcus aureus* (MRSA)	7 (4.5)
*Streptococcus spp*.	8 (5.2)
Anaerobic pathogen (other than *B. fragilis*)	8 (5.2)
*Stenotrophomonas maltophilia*	6 (3.9)
*Bacteroides fragilis*	4 (2.6)
Other *Enterococcus* spp.	4 (2.6)
Other *Staphylococcus* spp.	4 (2.6)
*Acinetobacter radioresistens*	1 (0.6)
*Haemophilus parainfluenzae*	1 (0.6)
*Lactobacillus* spp.	1 (0.6)
*Mycobacterium abscessus*	1 (0.6)
*Mycobacterium chelonae*	1 (0.6)
Polymicrobial infection	120 (77.4)
ID consult	139 (89.7)
Surgical intervention^*h*^	93 (60.0)

^*a*^ Data presented as number (%) or median (IQR) as appropriate.

^*b*^ Index organism(s) isolated from ≥1 source in some cases.

^*c*^ Mechanically ventilated > 48 h prior to pneumonia diagnosis.

^*d*^ Organisms with low susceptibility rates to ERV in the literature (15). Organisms present in polymicrobial culture in 8/9 cases. One patient received ERV monotherapy for the treatment of a *P. mirabilis *wound infection and experienced clinical success.

^*e*^ Nonsusceptibility to ceftriaxone, ceftazidime, or cefepime or positive identification of extended-spectrum β-lactamase.

^*f*^ Resistance to one or more carbapenem antibiotics or positive identification of carbapenemase.

^*g*^ Susceptibility determined using breakpoints established by the U.S. Food and Drug Administration (27).

^*h*^ Surgical intervention: incision and drainage (*n *= 39), debridement (*n *= 32), amputation (*n* = 5), IV catheter removal (*n* = 4), other invasive device removal (*n* = 2), valvular replacement (*n *= 1), and others (*n* = 33). Some patients underwent >1 surgical intervention.

^*i*^ Abbreviations: PJI, prosthetic joint infection; ERV, eravacycline; CRE, carbapenem-resistant Enterobacterales; ESBL: extended-spectrum β-lactamase-producing Enterobacterales; ID, infectious diseases.

Treatment characteristics are described in Table 3. ERV was initiated at a median of 117 h (IQR: 49–218) after the index culture was collected. A majority of patients (60%) received therapy with an antibiotic with activity against the index organism(s) prior to the initiation of ERV. The rationale for selecting ERV was primarily for consolidation of the antibiotic regimen (57%), presence or development of resistance to the first-line agent (12%), concerns relating to the safety of the first-line agent (12%), and/or double coverage for a CRE organism (10%). The median duration of ERV therapy was 6 days (IQR: 3.3–12.0), and 26% of patients received combination therapy (≥48 h) with another agent with Enterobacterales activity.

**TABLE 3:** Treatment characteristics (*n* = 155)^*a*^^,^^*d*^

Parameter	Value
ERV regimens	
1.5 mg/kg every 12 h	3 (1.9)
1 mg/kg every 12 h	142 (91.6)
1 mg/kg every 12 h × 1 day, then 1 mg/kg daily	4 (2.6)
1.5 mg/kg daily	1 (0.6)
0.5 mg/kg every 12 h	2 (1.3)
1 mg/kg daily	2 (1.3)
Not reported	1 (0.6)
Time to ERV, h (median, IQR)	117.0 (48.8–218.0)
ERV duration of therapy, days (median, IQR)	6.0 (3.3–12.0)
Rationale for ERV use	
Consolidation of the regimen	88 (56.8)
Resistance to the first-line agent	19 (12.3)
Safety concerns or intolerance to the first-line agent	18 (11.6)
Double CRE coverage	16 (10.3)
Lack of oral access	9 (5.8)
*C. difficile* activity/reduced *C. difficile* risk	6 (3.9)
Efficacy concerns with current therapy	6 (3.9)
Initial targeted treatment of polymicrobial infection	4 (2.6)
Empiric broad-spectrum coverage	2 (1.3)
Access or availability issues with the preferred agent	2 (1.3)
Unknown/not documented	2 (1.3)
Combination therapy^*b*^	40 (25.8)
Meropenem	13 (8.4)
Amikacin	5 (3.2)
Cefepime	5 (3.2)
Ceftazidime-avibactam	4 (2.6)
Ceftriaxone	3 (1.9)
Ciprofloxacin	3 (1.9)
Tobramycin	3 (1.9)
Ampicillin-sulbactam	2 (1.3)
Piperacillin-tazobactam	2 (1.3)
Ampicillin	1 (0.6)
Aztreonam	1 (0.6)
Ceftolozane-tazobactam	1 (0.6)
Ertapenem	1 (0.6)
Gentamicin	1 (0.6)
Levofloxacin	1 (0.6)
Meropenem-vaborbactam	1 (0.6)
Trimethoprim-sulfamethoxazole	1 (0.6)
Active anti-Enterobacterales therapy before ERV^*c*^	93 (60.0)
Cefepime	33 (21.3)
Meropenem	28 (18.1)
Piperacillin-tazobactam	24 (15.5)
Ertapenem	16 (10.3)
Ceftriaxone	14 (9.0)
Ceftazidime-avibactam	13 (8.4)
Piperacillin-tazobactam	11 (11.0)
Amikacin	4 (2.6)
Ciprofloxacin	4 (2.6)
Levofloxacin	4 (2.6)
Meropenem-vaborbactam	3 (1.9)
Tigecycline	3 (1.9)
Trimethoprim-sulfamethoxazole	3 (1.9)
Amoxicillin-clavulanate	2 (1.3)
Ampicillin	2 (1.3)
Ceftazidime	2 (1.3)
Gentamicin	2 (1.3)
Tobramycin	2 (1.3)
Ampicillin-sulbactam	1 (0.6)
Aztreonam	1 (0.6)
Ceftolozane-tazobactam	1 (0.6)
Cephalexin	1 (0.6)
Imipenem	1 (0.6)
Imipenem-relebactam	1 (0.6)
Moxifloxacin	1 (0.6)
Discharge disposition	
Home	64 (41.3)
Long-term acute care or skilled nursing facility	37 (23.9)
Death	26 (16.8)
Hospice	11 (7.1)
Rehab center	16 (10.3)
Not reported	1 (0.6)
Discharged on antibiotics	81 (52.3)
Discharged on ERV	31 (20.0)

^*a*^ Data presented as number (%) or median (IQR) as appropriate.

^*b*^ Active therapy: Demonstrated *in vitro* susceptibility.

^*c*^ Combination therapy: Antibiotic with Enterobacterales coverage administered for ≥48 h continuously while the patient received eravacycline.

^*d*^ Abbreviations: ERV, eravacycline; IQR, interquartile range; CRE, carbapenem-resistant Enterobacterales.

The primary outcome of clinical success was achieved in 84.5% of patients (Table 4). The 30-day all-cause mortality and infection-related mortality were 13.5% and 8.4%, respectively. Infection recurrence was low, with 2 patients (1.3%) experiencing microbiological recurrence at 30 days post-treatment. One of these patients was treated for an intra-abdominal infection, while the other was treated for a skin and soft-tissue infection. Both cases of microbiologic recurrence were accompanied by symptoms of infection and were subsequently treated with antibiotics. The 30-day hospital readmission rate was 16.9%, with four readmissions (2.6%) related to the index infection. Of these four readmissions, the index organism was isolated in culture in only a single case. Two patients received ERV on readmission. TEAEs attributable to ERV were rare. Eight patients (5.2%) experienced gastrointestinal intolerance, two (1.3%) experienced transaminitis, and one patient (0.6%) reported infusion site phlebitis. Acute kidney injury was observed in two (1.3%) patients. Notably, one of these patients was receiving combination therapy with cefepime, while the other was receiving ERV as monotherapy. Only a single incident of TEAE led to discontinuation of ERV (gastrointestinal intolerance).

**TABLE 4:** Clinical outcomes and tolerability (*n* = 155)^*a*^^,^^*g*^

Parameter	Value
Clinical success^*b*^	131 (84.5)
30-day all-cause mortality	21 (13.5)
30-day infection-related mortality	13 (8.4)
30-day microbiological recurrence^*c*^	2 (1.3)
Symptomatic	2 (1.3)
Treated with antibiotic(s)	2 (1.3)
Treatment-emergent resistance to ERV	1 (0.6)
Hospital readmission	
30-day hospital readmission	26 (16.9)
Related to index infection	4 (2.6)
New infection (unrelated to index infection)	7 (4.5)
Acute onset of a new condition	5 (3.2)
Acute exacerbation of a chronic condition	10 (6.5)
60-day hospital readmission^*d*^	18 (11.6)
Index organism isolated on readmission culture	1 (0.6)
ERV on readmission	2 (1.3)
Treatment-emergent adverse events	13 (8.4)
Gastrointestinal intolerance^*e*^	8 (5.2)
Transaminitis	2 (1.3)
Acute kidney injury^*f*^	2 (1.3)
Infusion site phlebitis	1 (0.6)
None	138 (89.0)
ERV discontinuation secondary to an adverse event	
Gastrointestinal intolerance^*e*^	1 (0.6)

^*a*^ Data are presented as number (%).

^*b*^ Clinical success: The resolution or improvement of infectious signs and symptoms including infection-related abnormal white blood cell count/temperature or as documented by the physician in clinical notes.

^*c*^ Microbiological recurrence: an isolate of the same bacteria (at species level) from the same culture source taken after a negative culture in an inpatient or ambulatory setting.

^*d*^ Patients with a 30-day readmission were muted from 60-day readmission total.

^*e*^ Gastrointestinal intolerance defined as nausea, vomiting, and/or diarrhea.

^*f*^ Acute kidney injury defined as an increase in serum creatinine by ≥0.5 mg/dL and ≥50% increase from baseline on two consecutive measurements. One patient was receiving combination therapy with eravacycline and cefepime. One patient was receiving eravacycline monotherapy.

^*g*^ Abbreviations: ERV, eravacycline.

In patients with infections caused by CRE, clinical success was achieved in 78.4% of patients, with 30-day all-cause and infection-related mortality occurring in 18.9% and 13.5% of patients, respectively (Table 5). No patients experienced microbiologic recurrence within 30 days from the end of treatment. Nine (24.3%) patients were readmitted to the hospital within 30 days of discharge from their index hospitalization. Among patients with infections caused by *Klebsiella pneumoniae*, *Escherichia coli*, and *Enterobacter cloacae*, clinical success rates were high (85.2%, 86.3%, and 79.4%, respectively), with comparable 30-day all-cause mortality across organisms (14.8%, 15.7%, and 11.8%, respectively). Additional organism-specific outcomes are described in Table 6.

**TABLE 5:** Clinical outcomes by the Enterobacterales resistance phenotype^*a*^^,^^*g*^

Parameter	CRE^*b*^ (*n* = 37)	ESBL^*c*^ (*n* = 31)	Non-CRE, non-ESBL (*n* = 87)
Clinical success^*d*^	29 (80.6)	28 (90.3)	74 (85.1)
30-day all-cause mortality	7 (18.9)	2 (6.5)	12 (13.8)
30-day infection-related mortality	5 (13.5)	1 (3.2)	7 (8.0)
30-day microbiological recurrence^*e*^	0 (0.0)	2 (6.5)	0 (0.0)
Hospital readmission			
30-day hospital readmission	9 (25.0)	4 (12.9)	13 (14.9)
60-day hospital readmission^*f*^	6 (16.2)	3 (9.7)	9 (10.3)

^*a*^ Data are presented as number (%).

^*b*^ Resistance to one or more carbapenem antibiotics or positive identification of carbapenemase.

^*c*^ Nonsusceptibility to ceftriaxone, ceftazidime, or cefepime or positive identification of extended-spectrum β-lactamase.

^*d*^ Clinical success: The resolution or improvement of infectious signs and symptoms including infection-related abnormal white blood cell count/temperature or as documented by the physician in clinical notes.

^*e*^ Microbiological recurrence: An isolate of the same bacteria (at species level) from the same culture source taken after a negative culture in an inpatient or ambulatory setting.

^*f*^ Patients with a 30-day readmission were muted from 60-day readmission total.

^*g*^ Abbreviations: CRE: carbapenem-resistant Enterobacterales; ESBL: extended-spectrum β-lactamase-producing Enterobacterales.

**TABLE 6:** Clinical outcomes by pathogen^*a*^

Parameter	*Klebsiella pneumoniae* (*n* = 54)	*Escherichia coli* (*n* = 51)	*Enterobacter cloacae* (*n* = 34)
Clinical success^*b*^	46 (85.2)	44 (86.3)	27 (79.4)
30-day all-cause mortality	8 (14.8)	8 (15.7)	4 (11.8)
30-day infection-related mortality	5 (9.3)	2 (3.9)	3 (8.8)
30-day microbiological recurrence^*c*^	0 (0.0)	1 (2.0)	0 (0.0)
Hospital readmission			
30-day hospital readmission	8 (14.8)	7 (13.7)	7 (20.6)
60-day hospital readmission^*d*^	8 (14.8)	6 (11.8)	5 (14.7)

^*a*^ Data are presented as number (%), minimum n = 20.

^*b*^ Clinical success: The resolution or improvement of infectious signs and symptoms including infection-related abnormal white blood cell count/temperature or as documented by the physician in clinical notes.

^*c*^ Microbiological recurrence: An isolate of the same bacteria (at species level) from the same culture source taken after a negative culture in an inpatient or ambulatory setting.

^*d*^ Patients with a 30-day readmission were muted from 60-day readmission total.

## DISCUSSION

In this study, we assess the real-world safety and efficacy of ERV for the treatment of mono- and polymicrobial infections involving Enterobacterales. High rates of clinical cure were observed, along with low rates of all-cause and infection-related mortality, supporting the efficacy of ERV in treating these infections. Notably, rates of clinical success were numerically similar across infections with *K. pneumoniae*, *E. coli*, and *E. cloacae*, organisms commonly isolated in intra-abdominal infections, reinforcing the utility of ERV in this infection type (17). The incidence of microbiological recurrence was limited, and index infection-related hospital readmission rates remained low. Additionally, the safety of ERV was demonstrated, as evidenced by the low rates of TEAEs observed in this cohort. The incidence of gastrointestinal intolerance was considerably lower than that previously reported for tigecycline in clinical trials, highlighting the clinical advantage of ERV compared to tigecycline with regard to tolerability (30).

Nearly a quarter of causative organisms in this study were CRE, with clinical success exceeding 80% in this subgroup. Many therapeutic regimens for the treatment of CRE outside of the urinary tract involve novel β-lactam-based regimens (4). While β-lactams are generally considered to have a favorable safety profile, neurotoxicity and nephrotoxicity are widely reported in the literature (31–33). Furthermore, a majority of β-lactam antibiotics are primarily renally eliminated, which may limit their appropriateness in patients with moderate-to-severe renal impairment. Most frequently, the mechanism of carbapenem resistance in Enterobacterales is β-lactamase-mediated. While novel β-lactam-β-lactamase inhibitor combination agents are active against the most common carbapenemases, no β-lactamase inhibitor approved by the FDA or European Medicines Agency directly inhibits metallo-β-lactamases. While the recently approved coformulation of aztreonam and avibactam maintains activity against the majority of metallo-β-lactamase-producing strains, it relies on the inability of these enzymes to hydrolyze aztreonam rather than inhibition of the enzymes themselves (8). This renders aztreonam-avibactam particularly susceptible to other mechanisms of resistance, such as penicillin-binding protein 3 (PBP3) mutations, which are increasing in prevalence (34). Failure of β-lactam-based therapies may lead to the use of agents with a greater risk of toxicity, including aminoglycoside- and polymyxin-based regimens. ERV appears to be a safe and effective drug for treating these recalcitrant infections due to its limited renal excretion, low nephrotoxicity potential, and efficacy independent of β-lactamase production (15).

Notably, a majority of patients included in our cohort had a polymicrobial index culture with *Enterococcus* spp., *Staphylococcus* spp., *Acinetobacter baumannii*, or other species associated with multidrug-resistant infections, in addition to Enterobacterales. These infections commonly require the administration of several concomitant antimicrobial agents to ensure adequate coverage of all infecting organisms. The administration of many broad-spectrum agents can disrupt gut microbiota, thereby increasing the likelihood for the development of *C. difficile* infection (35, 36). ERV is uniquely positioned as an effective agent for these types of infections due to its broad spectrum of activity and intrinsic efficacy against *C. difficile* (15). The predominant rationale for the utilization of ERV in this study was for consolidation of antimicrobial regimens, underscoring its utility in treating polymicrobial infections, particularly those involving multidrug-resistant organisms.

*Proteus mirabilis*, *Proteus vulgaris*, or *Providencia stuartii* were isolated in nine patients in this study despite these organisms exhibiting poor *in vitro* susceptibility to ERV in the literature (11, 15, 37). It is notable that these organisms were isolated in polymicrobial culture in eight of nine patients, and ERV was likely primarily targeting another co-infecting organism. One patient received ERV monotherapy for the treatment of a wound infection solely caused by *Proteus mirabilis* due to a history of severe allergy to other available agents. This patient achieved clinical success without microbiologic recurrence; however, caution is warranted when using ERV to treat infections caused by these organisms given their poor *in vitro* susceptibility.

This real-world study notably includes patient populations that were excluded from the IGNITE-1 and IGNITE-4 clinical trials, including patients with moderate-to-severe hepatic or renal impairment or those who are immunocompromised. In our cohort, 18% and 10% of patients had moderate-to-severe hepatic and renal dysfunction, respectively, and 12% of patients were immunocompromised. The inclusion of these patient populations did not appear to negate the demonstrated safety and efficacy of ERV in treating Enterobacterales infections.

The retrospective design of this study presents several inherent limitations. This study design is vulnerable to selection bias as patients who are receiving ERV have likely failed previous antibiotic regimens or have been determined to be at an increased risk for multidrug-resistant organisms. However, these scenarios may reflect ERV’s current place in therapy. Furthermore, in instances of polymicrobial infection, it is difficult to determine if the Enterobacterales organism was the primary driver of infection as opposed to other organisms isolated in culture. Additionally, due to variability in microbiological testing practices across institutions, detailed resistance mechanism characterization and minimum inhibitory concentrations (MICs) for ERV were not available for all isolates included in this study. While tigecycline susceptibility is closely correlated with that of ERV (38), tigecycline MICs were only available in 43% of isolates. Lastly, this study lacked a comparator arm given its descriptive nature; therefore, definitive conclusions of the efficacy and safety of ERV relative to other agents cannot be determined.

This real-world study supports the safe and effective use of ERV for the treatment of Enterobacterales infections, including CRE, adding to our armamentarium of antimicrobial agents designed to combat this growing threat. Future prospective, comparative studies are needed to validate these findings.
